# Distribution of Midbrain Cholinergic Axons in the Thalamus

**DOI:** 10.1523/ENEURO.0454-19.2019

**Published:** 2020-01-06

**Authors:** Icnelia Huerta-Ocampo, Husniye Hacioglu-Bay, Daniel Dautan, Juan Mena-Segovia

**Affiliations:** 1 Center for Molecular and Behavioral Neuroscience, Rutgers University, Newark, NJ 07102; 2 MRC Anatomical Neuropharmacology Unit, University of Oxford, Oxford OX1 3TH, United Kingdom; 3 Department of Anatomy, School of Medicine, Marmara University, 34854 Istanbul, Turkey

**Keywords:** cholinergic innervation, conditional tracing, laterodorsal tegmental nucleus, pedunculopontine nucleus

## Abstract

Cholinergic transmission is essential for adaptive behavior and has been suggested to play a central role in the modulation of brain states by means of the modulation of thalamic neurons. Midbrain cholinergic neurons from the pedunculopontine nucleus (PPN) and the laterodorsal tegmental nucleus (LDT) provide dense innervation of the thalamus, but a detailed connectivity mapping is missing. Using conditional tracing of midbrain cholinergic axons in the rat, together with a detailed segmentation of thalamic structures, we show that projections arising in PPN and LDT are topographically organized along the entire extent of the thalamus. PPN cholinergic neurons preferentially innervate thalamic relay structures, whereas LDT cholinergic neurons preferentially target thalamic limbic nuclei. Moreover, both PPN and LDT provide a dense innervation of the intralaminar thalamic nuclei. Notably, we observe a differential synaptic density that functionally dissociates between PPN and LDT innervation. Our results show that midbrain cholinergic neurons innervate virtually all thalamic structures and this innervation is functionally segregated.

## Abstract (precis)

Cholinergic transmission is essential for adaptive behavior and has been suggested to play a central role in the modulation of brain states by means of the modulation of thalamic neurons. Midbrain cholinergic neurons from the pedunculopontine nucleus (PPN) and the laterodorsal tegmental nucleus (LDT) provide dense innervation of the thalamus, but a detailed connectivity mapping is missing.

## Significance Statement

The cholinergic midbrain provides dense innervation to the thalamus and modulates its activity across different behaviors. However, the magnitude of this projection and the precise distribution of these axons in relation to different functional thalamic regions remain poorly understood. Here, we selectively labeled cholinergic axons arising in the pedunculopontine nucleus (PPN) and the laterodorsal tegmental nucleus (LDT) using conditional viral tracing. We reveal that PPN and LDT cholinergic axons are topographically organized and provide a segregated innervation of thalamic structures. PPN neurons preferentially innervate relay thalamic nuclei, whereas LDT neurons preferentially innervate thalamic limbic nuclei. Our results suggest that neurons along the functional domains of the cholinergic midbrain have a differential impact on thalamic circuits.

## Introduction

The thalamus is a complex, functionally-heterogeneous brain region that modulates the activity of neural circuits in the cortex and the basal ganglia (Smith et al., 2004, 2011; Haber and Calzavara, 2009; Sherman, 2016; Halassa and Kastner, 2017; Wolff and Vann, 2019). It receives dense cholinergic innervation that arises mainly in the midbrain and, to a lesser extent, in the basal forebrain (Hallanger et al., 1987; Steriade et al., 1987a,b, 1988; Parent et al., 1988). The midbrain cholinergic innervation has been considered as diffuse, widespread and non-specific, targeting thalamic nuclei that in turn have non-specific neocortical projections (Scheibel and Scheibel, 1967; Groenewegen and Berendse, 1994). Such projections have been traditionally associated with the induction and maintenance of tonic activation mechanisms in thalamocortical systems associated with the desynchronization of the electroencephalogram that occurs during waking and REM sleep (Steriade et al., 1990). More recently, accumulating experimental evidence has led to the proposal that the thalamus has a role in working memory and instrumental conditioning, largely sustained by its anatomic connections with structures implicated in these functions, such as the basal ganglia (Gabriel et al., 1989; Bradfield et al., 2013; Parnaudeau et al., 2013, 2015; Alcaraz et al., 2018).

The pattern of distribution of cholinergic axons in the thalamus has been studied in the cat, rat and monkey using a combination of conventional retrograde neuronal tracers and immunohistochemistry for choline acetyltransferase (ChAT; Sofroniew et al., 1985; Hallanger et al., 1987; Paré et al., 1988; Steriade et al., 1988; Bolton et al., 1993), but either the large volume of tracers injected was not confined to a single thalamic structure, or the number of injections only targeted a low sample of thalamic nuclei. While in other studies some detailed maps of retrograde thalamic midbrain innervation have been created, the neurochemical nature of this innervation was not established (Krout et al., 2002). In turn, injections of the anterograde tracer PHA-L into the midbrain region that contains cholinergic neurons, combined with immunohistochemistry to confirm the cholinergic nature of their projections, have shown dense labeling in thalamic structures. However, technical constraints in these studies allowed only a general anatomical characterization and the density of these projections was not quantified (Satoh and Fibiger, 1986; Hallanger and Wainer, 1988; Cornwall et al., 1990). Complementarily, detailed descriptions of cholinergic innervation have been reported for some thalamic nuclei based on the distribution of ChAT-immunopositive fibers (Stichel and Singer, 1985; Hallanger et al., 1987; Fitzpatrick et al., 1989; Heckers et al., 1992; Kitt et al., 1994; Parent and Descarries, 2008), but because cholinergic axons may arise in different sources (i.e., midbrain and basal forebrain), it was not possible to discern their origin. Thus, the meticulous experiments described above have established that cholinergic neurons of the midbrain innervate the thalamus by (1) reporting the localization of cholinergic and non-cholinergic thalamic-projecting neurons of the midbrain and (2) reporting the pattern of connectivity of cholinergic axons in some of the thalamic nuclei. However, detailed information about the distribution and density of axonal projections to individual thalamic nuclei associated with the different functional regions in the cholinergic midbrain is still missing.

Cholinergic neurons of the midbrain are located in two functionally-different structures: the pedunculopontine nucleus (PPN) and the laterodorsal tegmental nucleus (LDT). These neurons possess long-range axonal projections giving rise to many collaterals that reach a variety of targets in the thalamus, basal ganglia, and basal forebrain (Mena-Segovia, 2016). Neurons of the PPN and the LDT include, in addition to the cholinergic ones, glutamatergic and GABAergic. Of the non-cholinergic neuronal populations, glutamatergic neurons have also been shown to project to the thalamus (Barroso-Chinea et al., 2011), highlighting the importance of discerning the neurochemical nature of the midbrain projections over different regions of the thalamus. Furthermore, PPN and LDT maintain connectivity with forebrain targets that are integrated into distinct functional circuits, where PPN preferentially targets motor circuits and LDT preferentially targets limbic circuits, including thalamic regions (Satoh and Fibiger, 1986; Hallanger and Wainer, 1988; Cornwall et al., 1990). To fully understand how acetylcholine impacts on thalamic circuits, it becomes then critical to obtain a detailed map of the connectivity of PPN and LDT cholinergic neurons across different thalamic domains.

Here, we used AAV-mediated conditional tracing in ChAT::Cre^+^ rats to quantify the density of cholinergic innervation provided by the PPN and LDT to 47 individually-identified thalamic nuclei. Our results demonstrate that there is heterogeneity and selectivity in the innervation provided by the cholinergic midbrain.

## Materials and Methods

### Animals

All experimental procedures were performed on adult male and female ChAT::Cre^+^ rats (Witten et al., 2011). Rats were maintained on a 12/12 h light/dark cycle (light on 7:00 A.M.) and *ad libitum* access to water and food. All procedures were performed in accordance with the Society for Neuroscience policy on the use of animals in neuroscience and were approved by the Home Office or the Institutional Animal Care and Use Committee, in compliance with the Animals (Scientific Procedures) Act, 1986 (United Kingdom) or the Guide for the Care and Use of Laboratory Animals (Department of Health and Human Services), respectively.

### Stereotaxic injections

Surgeries were performed under deep isoflurane anesthesia (2% in O_2_; Isoflo; Schering-Plow). For the analysis of distribution and mapping of cholinergic axons into the distinct thalamic nuclei, ChAT::Cre^+^ rats were injected with an adeno-associated virus serotype 2 (AAV2) carrying the fusion gene for yellow fluorescent protein (AAV2–EF1a-DIO-eYFP; Gene Therapy Center Virus Vector Core, University of North Carolina). For the identification of axon terminals of cholinergic neurons in the thalamus, ChAT::Cre^+^ rats were injected with an AAV-FLEX-Synaptophysin-mRuby virus (Stanford Vector Core). AAVs were injected in the caudal part of the PPN (400 nl over 10 min; from bregma in mm: AP, −7.8; ML, +1.8; DV, −6.5 ventral of the dura; *n* = 3), or the LDT (300 nl over 10 min; from bregma in mm: AP, −8.5; ML, +0.9; DV, −6.0 ventral of the dura; *n* = 3; Paxinos and Watson, 2007). Initial injections were aimed to also include a group targeting the rostral part of the PPN (*n* = 3), but the pattern of axonal labeling in thalamic structures was not consistent between animals, particularly due to the technical difficulties of selectively reaching the most rostral end of the nucleus (also known as pars dissipata), where the number of cholinergic neurons is very small. All injections were made using designated 1-μl syringes (SGE Analytical Science) at a rate of 40 nl/min and a postinjection diffusion time of 5 min. Approximately six weeks after the virus injection, the rats were given a lethal dose of pentobarbital (200 mg/kg, i.p.) and perfused transcardially with 0.05 M PBS, pH 7.4, followed by 300 ml of 4% w/v paraformaldehyde in phosphate buffer (0.1 m, pH 7.4) containing 0.1% glutaraldehyde (TAAB Laboratories). Brains were stored in PBS with 0.05% azide at 4°C until sectioning.

### Immunohistochemistry

Coronal sections of 50-μm thickness were obtained and collected in PBS, using a vibratome (VT1000S; Leica) and organized in six series. For each brain, the site of injection was verified and only those with on-target injections were processed further. All the incubations were done in PBS containing 0.3% v/v Triton X-100 (Sigma; Triton-PBS). All selected sections were blocked for 2 h at room temperature (RT) while shaking in Triton-PBS containing 10% v/v of normal donkey serum (NDS; Jackson ImmunoResearch). Next, they were incubated overnight in either a rabbit anti-GFP antibody coupled with a 488 fluorophore (1:1000, Invitrogen, A-21311) or a rat anti-GFP (1:1000, Nacalai tesque, 04404-84) followed by 6-h incubation in an anti-rat-488 (1:500; Jackson ImmunoResearch, 712-546-153) in Triton-PBS containing 1% of NDS. To visualize the cytoarchitecture of thalamic nuclei and delineate their borders to map the projections arising from each of the injected midbrain regions, we incubated the sections subsequently for 3 h at RT in NeuroTrace (1:500; Life Technologies, N21479, N21482), a blue or red fluorescent Nissl stain containing Triton-PBS for 3 h. We chose fluorescence Nissl stain over Neu-N immunofluorescent labeling as it produced a clearer labeling and lower background, which allowed us to define more clearly the borders of the thalamic nuclei. After several washes, the fluorescently-labeled sections were mounted on glass slides using Vectashield and examined in a fluorescent (ImagerM2; Zeiss) or confocal (LSM-510; Zeiss and FV-2000; Olympus) microscope. The brightness and contrast of the captured images were adjusted in Photoshop (Adobe Systems).

### Distribution of cholinergic axons

Sections from each group (PPN and LDT) were analyzed separately. We ran a pilot experiment in which we initially analyzed 18 sections per animal to determine the distribution of axons in all 47 thalamic structures. From this initial sample, we selected five levels along the anteroposterior axis (AP –1.7, –2.5, –3.2, –4.1, and –4.8 mm) that represented all nuclei belonging to the anterior, midline, ventral, and posterior thalamus, and constituted the number of AP levels used for further analysis. For each section analyzed, we manually outlined the borders of each thalamic structure using the Nissl labeling at 10× magnification. A grid of 60 × 80 μm was superimposed using StereoInvestigator (MicroBrightField) and was used to quantify the distribution of labeled axons in each thalamic structure along the five sections selected for analysis. Each grid containing at least one YFP-positive-labeled axon was marked as positive, see (Dautan et al., 2014) . This analysis provides information about the distribution of axons but not about the axonal density. The data are represented as the area of each thalamic nucleus occupied by labeled axons and is expressed as the percentage of innervation of the total grid area.

### Quantification of synaptophysin innervation

To quantify the relative synaptic incidence of cholinergic terminals in thalamic nuclei, we selected those thalamic structures receiving the highest and lowest distribution of axons originated in the PPN and LDT. We included the Rt, DLG, MG, PO, LPMR, VPL, Pf, CM, MD, AM, and PV nuclei (for abbreviations, see Table 1). Sections containing the selected nuclei were scanned in a confocal microscope using a 40× magnification objective at 10-, 20-, and 30-μm-depth along the *z*-axis. The area selected for scanning corresponded to the area with the densest number of particles observed for each structure. The number of synaptophysin particles was analyzed in ImageJ using the particle analysis in-built function.

**Table 1.** Summary of abbreviations

Abbreviation	Structure	Abbreviation	Structure
AD	Anterior dorsal	MDL	Mediodorsal lateral
AM	Anterior medial	MDM	Medial dorsal medial
AMV	Anterio medio-ventral	MG	Medial geniculate
Ang	Angular	MGD	Medial geniculate dorsal
AV	Anterioventral	MGV	Medial geniculate ventral
AVDM	Anterioventral dorsomedial	MHb	Medial habenula
AVVL	Anterioventral ventrolateral	OPC	Oval paracentral
CL	Central lateral	PC	Paracentral
CM	Central medial	Pf	Parafascicular
DLG	Dorsolateral geniculate	PO	Posterior nucleus
IAD	Interanterio-dorsal	PPN	Pedunculopontine
IAM	Interanterio-medial	PT	Paretenial
ID	Intermediodorsal	PV	Paraventricular
LHb	Lateral habenula	PV Ant	Paraventricular anterior
LHbM	Lateral habenula medial	PVPo	Paraventricular posterior
LHbL	Lateral habenula lateral	Re	Reuniens
LDDM	Lateral dorsal dorsomedial	Rh	Rhomboid
LDVL	Lateral dorsal ventrolateral	Rt	Reticular thalamic
LDT	Laterodorsal tegmental	VA	Ventral anterior
LPLC	Lateral posterior laterocentral	VL	Ventral lateral
LPLR	Lateral posterior laterorostral	VLG	Ventrolateral geniculate
LPMC	Lateral posterior mediocentral	VM	Ventral medial
LPMR	Lateral posterior mediorostral	VPL	Ventral posterolateral
MD	Mediodorsal	VPM	Ventral posteromedial
MDC	Mediodorsal central	VPPC	Ventral posterior parvicellular

## Results

**Figure 1.**
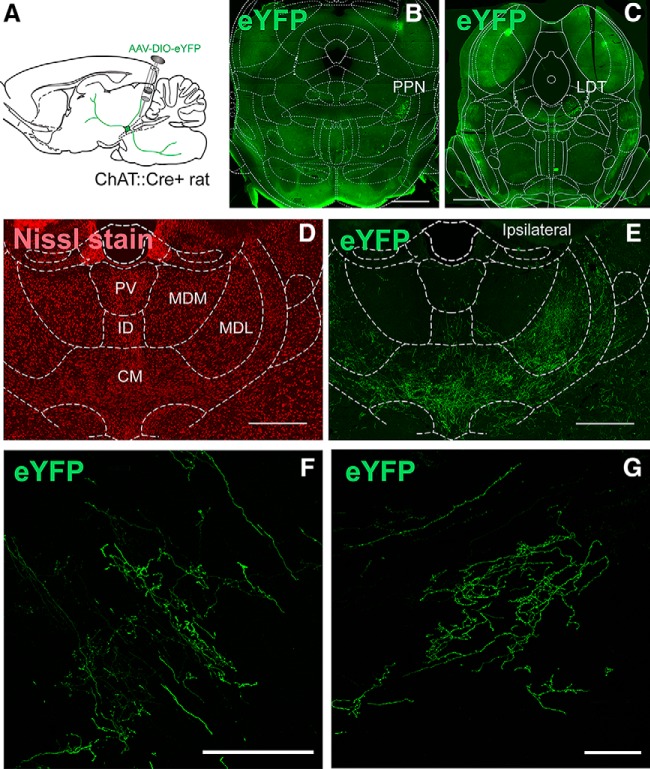
Analysis of the distribution of midbrain cholinergic axons in the thalamus. ***A***, Experimental strategy: AAV2–EF1a-DIO-eYFP was injected into the caudal portion of the PPN or the LDT. ***B***, ***C***, YFP-labeled neurons were located at the level of the PPN (***B***) or the LDT (***C***). ***D***, ***E***, Selected sections were Nissl-stained to delimit the borders of individual thalamic nuclei and were used to determine the distribution of axons (***E***) on each nucleus. ***F***, ***G***, Representative coronal confocal images showing the pattern of cholinergic axonal thalamic projections arising in the PPN (***F***) and LDT (***G***). Scales bars: 1 mm (***B***, ***C***), 500 μm (***D***, ***E***), and 100 μm (***F***, ***G***).

**Figure 2.**
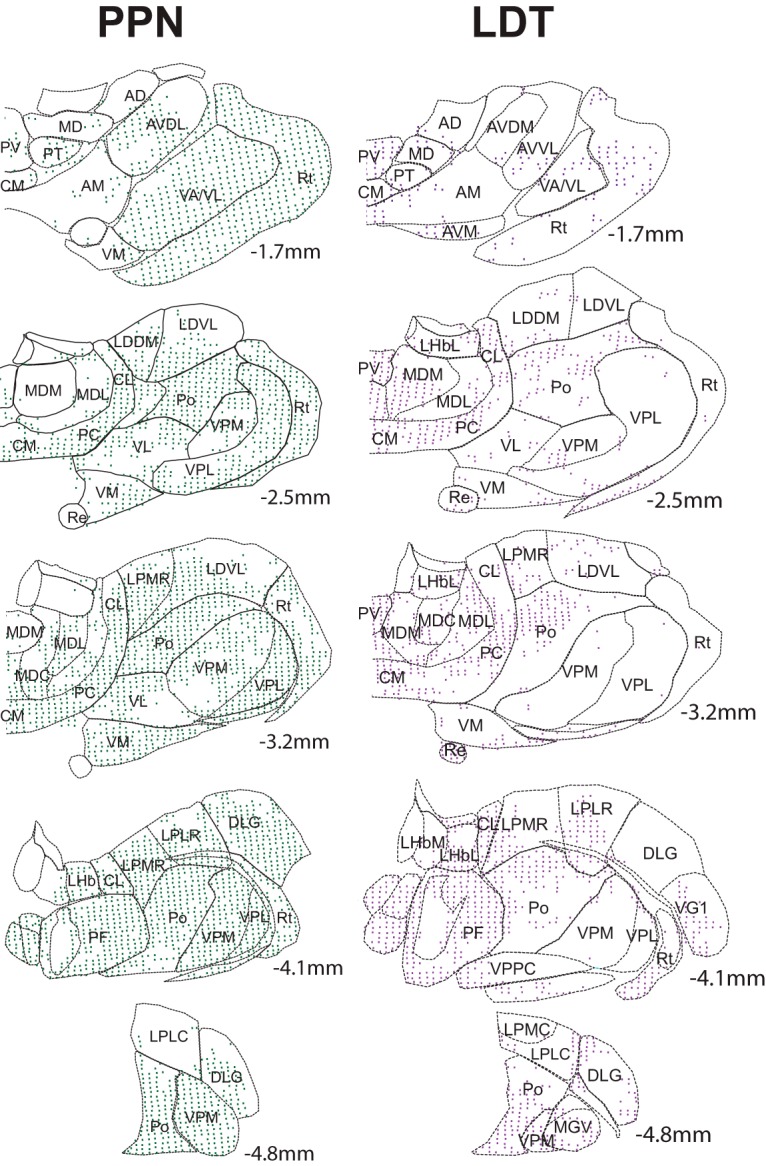
Mapping of midbrain cholinergic axons in individual thalamic nuclei. The spatial distribution of cholinergic axons arising in the PPN (left) or the LDT (right) on each thalamic nucleus was mapped in five selected, Nissl-stained coronal sections along the rostro-caudal axis. Each colored dot represents a 60 × 80 μm grid that contained YFP-positive axons.

Conditional YFP transduction in the PPN/LDT of ChAT::Cre^+^ rats has been reported to produce highly selective labeling of cholinergic neurons (Dautan et al., 2016). The labeled axons of transduced cholinergic neurons from the PPN (Fig. 1*A*,*B*) and LDT (Fig. 1*C*) gave rise to widespread midbrain and forebrain projections in agreement with previous reports (Satoh and Fibiger, 1986; Hallanger and Wainer, 1988; Cornwall et al., 1990; Dautan et al., 2014, 2016). From all these projections, the main target was the thalamus, where they appeared to innervate all modalities of thalamic nuclei. The axons were strongly labeled, they extended across various thalamic regions and their distribution was correlated with the area of injection of the cholinergic midbrain. To delimit the localization and distribution of axons within the thalamus, we used a Nissl fluorescent staining which allowed us to outline the borders of the distinct thalamic nuclei using classical anatomical descriptions that have been reported in the literature (Fig. 1*D*,*E*; Jones, 2007). The distribution of axons was logged into the thalamic maps across five anteroposterior levels (AP –1.7, –2.5, –3.2, –4.1, and –4.8 mm; Fig. 2), thus covering all thalamic nuclei located in the anterior, midline, ventral, and posterior thalamus. YFP-labeling revealed that midbrain cholinergic axons are highly arborized, follow long and tortuous trajectories, and most of them are characterized by a large number of varicosities (Fig. 1*F*,*G*). In many instances, axons traveled across different thalamic nuclei and the innervation extended across the borders of neighboring structures, whereas in other cases, the distribution of axons was restricted to one particular thalamic region, and therefore, it was possible to discern the exact limits of this innervation to one specific nucleus. Thus, this innervation was heterogeneous and followed a specific topographic organization. In addition, midbrain cholinergic afferent projections were found to arise predominantly from the ipsilateral midbrain, although important contralateral projections were also observed (Fig. 1*E*).

### Innervation of the thalamus by cholinergic axons arising in the PPN

**Figure 3.**
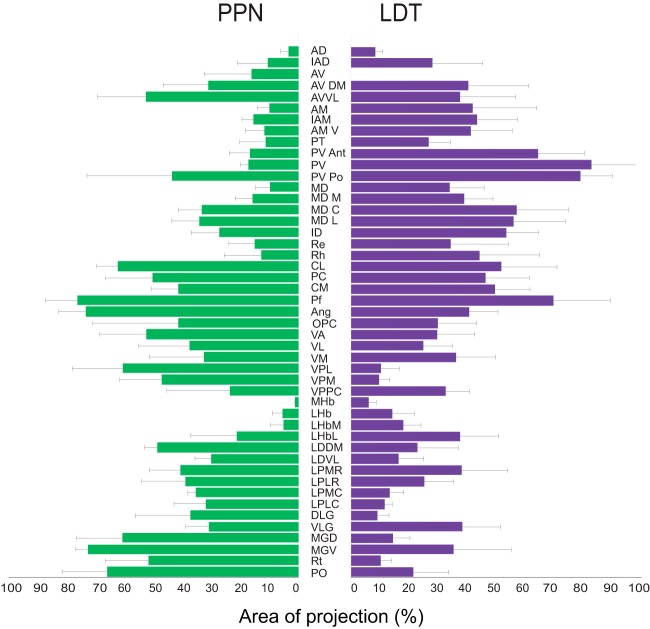
Axonal distribution of PPN and LDT cholinergic axons in the thalamus. Histogram showing the comparison of the distribution density of PPN (*n* = 3) and LDT (*n* = 3) YFP-transduced cholinergic axons. Data are expressed as the percentage of the total area occupied by labeled axons (defined by the counting grid) for each of the thalamic structures (mean ± SEM).

Injections in the PPN were initially aimed to separately transduce the rostral and caudal segments of the PPN. However, cholinergic neurons of the rostral PPN are sparse (pars dissipata) and tended to produce lower-density, inconsistent axonal labeling in the thalamus. While a few differences in the innervation between the rostral and caudal portions were observed (cholinergic axons of the rostral PPN more prominently innervate the AD, AMV, LHb, DLG, and VLG), the axon distribution followed an overall similar pattern. For this reason, we restricted our analysis to the caudal portion of the nucleus (Fig. 1*B*). Following six weeks of transduction, we observed areas of dense innervation that distributed widely throughout the thalamus (Fig. 2, left). A prominent distribution of axons was present in the caudal intralaminar nuclei (77.9% of all grid area occupied by cholinergic axons, hereafter shown as %), which in rodents corresponds to the lateral and medial parts of the parafascicular nucleus (Pf; Figs. 2, 3). Axons also distributed importantly in the rostral intralaminar group, particularly in the central medial (CM; 42.4%), paracentral (PC; 51.4%), and central lateral (CL; 63.7%) nuclei, from which the latter showed a preferential distribution. Areas of important innervation were observed, as expected, in nuclei involved in the relay of primary sensations such as the nucleus ventral posterolateral (VPL; 61.9%), ventral posteromedial (VPM; 48.3%), ventral anterior (VA; 53.7%), posterior nucleus (PO; 67.5%) as well as both portions of the lateral (LG; 31.5% and 38%) and medial geniculate (MG; 72.2% and 62%). Among these nuclei, the ventral portion of the MG receives preferential innervation. Thalamic associative nuclei were moderately innervated by the PPN. Within this category of nuclei, the laterodorsal dorsomedial nucleus (LDDM; 49.8%) tends to receive stronger innervation than the laterodorsal ventrolateral nucleus (LDVL; 30.8%). The associative lateroposterior complex, which included the lateral posterior mediorostral (LPMR; 41.6%), lateral posterior laterorostral (LPLR; 39.8%), lateral posterior mediocentral (LPMC; 36.3%) and lateral posterior laterocentral (LPLC; 32.7%) all received a similar pattern of labeling. Distinctively, the reticular nucleus (Rt) was found to be densely innervated by the PPN (52.9%), in contrast to the low innervation provided by the LDT (10.4%). The innervation provided to the Rt was present along the entire rostro caudal extent. Nuclei located in the midline, which have been categorized as the limbic thalamus, i.e., anterior dorsal, anterior medial, and paraventricular, received notably less innervation from the PPN.

### Innervation of the thalamus by cholinergic axons arising in the LDT

Injections in the LDT (Fig. 1*C*) gave rise to high-density projections that were mostly concentrated along the midline thalamus. Similar to the PPN injections, cholinergic LDT axons were highly collateralized, and possessed a high number of varicosities in their target nuclei (Fig. 1*G*). Within this region, they preferentially distributed along the paraventricular nucleus in its anterior (PVa; 65.4%), middle (PV; 84.2%), and posterior portions (PVp; 80.3%; Figs. 2, right, 3). Labeled fibers densely distributed ventral to the midline of the intermediodorsal nucleus (IMD) and extended laterally to densely innervate the central (MDc; 58%) and lateral portions (MDl; 56.9%) of the mediodorsal nucleus (MD). Ventrally along the midline, LDT axons distributed moderately in the rostral intralaminar nuclei, including CM and extending to the PC and CL nuclei, whereas labeled fibers were heavily distributed caudally to both the lateral and medial parts of the Pf (Fig. 3, 70.8%). Other limbic nuclei were also densely innervated, such as the rhomboid (13.1%), reuniens (15.4%) and various nuclei of the anterior thalamus.

Altogether, these data reveal that there is a topographic organization of the cholinergic midbrain projections to the distinct thalamic nuclei, where the PPN provides its densest innervation to associative and relay thalamic nuclei, whereas the LDT preferentially innervates midline nuclei involved in limbic circuits. Notably, although PPN and LDT seem to target functionally different thalamic nuclei, both midbrain structures provide a prominent innervation of the rostral and caudal intralaminar nuclei, where neurons that project to the striatum are predominantly located (Figs. 2, 3).

### The cholinergic midbrain provides synaptic innervation the thalamus

**Figure 4.**
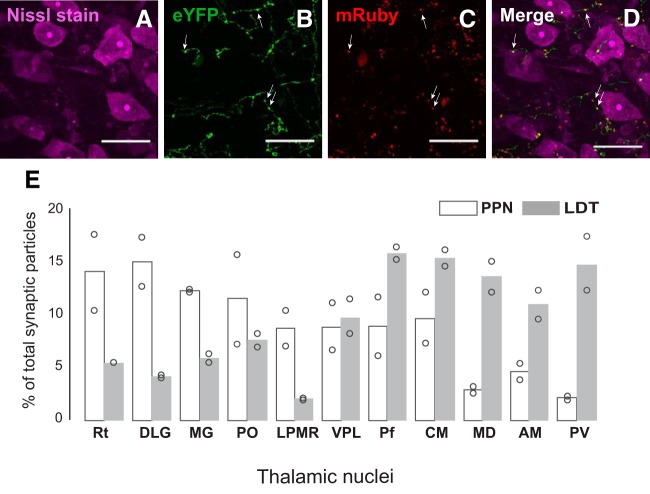
Quantification of midbrain cholinergic synapses in the thalamus. ***A–D***, Nissl-stained sections from animals injected with AAV-FLEX-Synaptophysin-mRuby were used to quantify, in YFP-labeled axons (**B**) and their varicosities (arrows), the number of synaptophysin particles (***C***, ***D***) across selected thalamic nuclei. ***E***, The number of synaptophysin particles was determined on 11 selected thalamic nuclei and is represented as the percentage of synaptophysin particles in each nucleus out of the total number of particles found in all selected nuclei included in this analysis. The values of the two animals included in the analysis (circles) are shown individually. Scale bar: 25 μm.

Next, we aimed to determine whether the data obtained from the distribution of midbrain cholinergic axons also reflected the density of synaptic connectivity in thalamic structures. To determine whether PPN and LDT axons establish synapses in the thalamus, we tagged the presynaptic protein synaptophysin with a fluorescent reporter (AAV-FLEX-mGFP-2A-Synaptophysin-mRuby) in midbrain cholinergic axon terminals using a similar viral strategy as above. We counted the relative number of mRuby-puncta on a selection of thalamic nuclei (Rt, DLG, MG, Po, LPMR, VPL, Pf, CM, MD, AM, and PV) which was based on the distribution of PPN and LDT axons (see Methods; Fig. 3). We detected, in Nissl co-stained sections (Fig. 4*A–D*), the presence of YFP-labeled axons with multiple varicosities along their trajectory (Fig. 4*B*) and therefore the presence of mRuby-tagged synaptic particles (Fig. 4*C*,*D*). The analysis of the number of fluorescently-labeled puncta reporting the presence of synaptophysin revealed that, in the case of the LDT, the thalamic nuclei with the densest distribution of axons also possessed the largest density of synaptic terminals formed by cholinergic neurons, and they were located in the paraventricular and caudal intralaminar nuclei (Fig. 4*E*). Notably, nuclei with a lower distribution of axons, such as the CM and mediodorsal, showed a similar number of synaptic terminals to those nuclei that showed the highest distribution of axons, i.e., the paraventricular and Pf, thus suggesting that LDT axons provide a dense innervation in some thalamic structures despite the more limited distribution of axons. Relay thalamic nuclei such as the DLG, MG, and Po, characterized by containing a low distribution of LDT axons, also displayed a low number of synaptic terminals.

In the case of the PPN, our results show that the number of synaptic particles in the Pf, the structure which has the highest distribution of axons, was lower than the number of puncta observed in the LG and Rt (Fig. 4*E*). Thalamic nuclei with a low distribution of axons, such as the PV and MD, contained a low number of synaptic terminals, suggesting that they may be axons en-passage giving a low number of synapses. We compared the number of synaptophysin puncta in all selected structures between PPN and LDT groups and found that LDT axons produce a significantly larger number of labeled puncta than PPN [PPN (200.40 ± 28.78) and LDT (339.27 ± 51.86), one-way ANOVA *F*_(1,20)_ = 7.14, *p* = 0.0151], suggesting that the LDT produces a denser synaptic innervation in the thalamic structures with higher distribution of cholinergic axons.

## Discussion

Our findings provide the first detailed topographic mapping of identified cholinergic midbrain projections in the entire thalamus. We observed that cholinergic projections arising in the anterior (PPN) and posterior (LDT) midbrain have an extensive distribution within the thalamus and reach virtually every thalamic nucleus. Nevertheless, the analysis of the distribution of axons and the synaptic density shows that the innervation is specialized and highly heterogeneous, as different thalamic structures were identified to receive a preferential innervation from either the PPN or the LDT. Thus, the PPN seems to mainly target relay thalamic nuclei, whereas the LDT mainly innervates midline thalamic nuclei, which are involved in limbic functions. Notably, both midbrain regions provide dense innervation to the intralaminar nuclei, which constitute a major source of excitatory inputs to the striatum and contribute to the diffuse innervation of several cortical regions, thus highlighting the role of cholinergic midbrain neurons in the modulation of this key thalamic hub.

### PPN and LDT coincident innervation

Our data show that both regions of the cholinergic midbrain innervate all components of the intralaminar thalamus. Densely labeled axons were distributed in the CM and extended to the PC and CL. This innervation was homogenous within these nuclei and distributed along the rostro-caudal axis. Among the intralaminar nuclei, the Pf showed the greatest distribution of axons after injections in both midbrain regions. Nevertheless, in the case of the PPN, we did not find a correlation between area of innervation and number of synapses labeled with synaptophysin. In contrast, LDT axons reaching the Pf seem to possess high density of synapses. The intralaminar thalamic nuclei were considered for a long time as “non-specific relay nuclei” with widespread non-specific connections with the cortex. However, this notion has changed with the demonstration that each individual nucleus of the rostral and caudal intralaminar nuclei projects to limited and specific cortical regions, as well as to specific functional subregions of the basal ganglia (Berendse and Groenewegen, 1991). The CL, PC and CM innervate the dorsolateral and most medial part of the striatum, whereas the Pf preferentially innervates the mediolateral, ventrolateral and ventromedial striatum as well as the nucleus accumbens. Altogether, the intralaminar complex innervates all functional domains of the striatum. Regarding their cortical projections, they have been reported to be restricted to specific layers of prefrontal areas, cingulate cortex, insular cortex, and prelimbic cortical areas among others. The intralaminar nuclei have been classically conceived as a crucial link for transmitting to the cerebral cortex the increased activity of midbrain neurons during activated states of vigilance. Thus, cholinergic midbrain neurons are in a position to influence striatal and cortical activity through the modulation of the activity of the intralaminar thalamic neurons that project to the striatum and cortex.

### Specialized thalamic innervation by the PPN and the LDT

Cholinergic neurons of the PPN innervate parts of the thalamus involved in the transmission of unimodal sensory information such as the MG and LG, which relay acoustic and visual information to the distinct cortical layers, respectively (Steriade et al., 1988). Other principal nuclei that are also importantly innervated by the PPN are the VPM and VPL, both of which relay specific somatosensory information. In addition, the PPN targets associative nuclei such as the LD and LP. Distinctively, we found that the Rt receives dense innervation arising from the PPN but not from the LDT. Previous anatomical experiments using anterograde and retrograde tracers in the rat have reported that innervation from brainstem neurons to the Rt is virtually absent or at least extremely sparse (Berry et al., 1986), and that most of the cholinergic innervation to the Rt arises in the basal forebrain. However, a more recent study using a ChAT-cre mouse model shows that PPN, LDT and the basal forebrain contribute similarly to the innervation of the Rt (Sokhadze et al., 2019). The physiological evidence further supports the functionality of these midbrain projections and suggests that is both inhibitory and cholinergic (Ben-Ari et al., 1976; Dingledine and Kelly, 1977). However, the synaptic mechanisms underlying cholinergic transmission in the Rt are not well understood. Rt neurons represent the only GABAergic thalamic nucleus, they form an essential part of the circuits that link the thalamus to the cortex and are involved in the generation of spindle oscillations during periods of transition from waking to sleep (Steriade et al., 1985). The existence of a dense innervation of Rt neurons by midbrain cholinergic afferents could provide a neuronal substrate for the disruption of synchronized spindle oscillatory activity on arousal and REM sleep, as classically established (Steriade et al., 1985, 1987a). In addition, as Rt neurons maintain topographic connectivity not only with thalamocortical neurons but also with almost all thalamic nuclei, apart from the anterior nuclei (Jones, 1975; Guillery et al., 1998), cholinergic PPN neurons may be modulating indirectly, via the Rt, the activity of various thalamic nuclei in addition to thalamocortical circuits.

In contrast to the PPN, cholinergic neurons of the LDT provide a clear preferential innervation of those thalamic nuclei that have been shown to play an important role in limbic functions. These projections include the PV and many other nuclei that lie in the midline thalamus and that extend in the rostro-caudal axis. This collection of midline nuclei shares common features, such as being recipient of a dense peptidergic innervation and their connectivity with the prefrontal cortex and ventromedial striatal regions (in turn associated with these same cortical areas). The PV is a major source of thalamic innervation to the nucleus accumbens, extending to the bed nucleus of the stria terminalis, and from the ventral striatum to the central nucleus of the amygdala, thus regulating neuronal circuits involved in motivation, reward and emotional mechanisms (Li and Kirouac, 2008; Kirouac, 2015). Also located in the midline there is an important distribution of axons in the central and lateral parts of the MD nucleus, which has been involved in cognitive processes such as associative learning and decision-making (Corbit et al., 2003; Mitchell et al., 2007; Parnaudeau et al., 2013) due to its extensive cortico-thalamo-cortical connections with the prefrontal cortex (Goldman-Rakic and Porrino, 1985; Groenewegen, 1988). The rhomboid nucleus as well as the anteroventral and anteromedial complexes, which are involved in navigation and spatial working memory in rodents, also display a large distribution of LDT labeled axons. Similar to the PPN, the LDT is innervating the rostral intralaminar nuclei including the CL, CM, and PC.

In conclusion, the differences in axonal density that PPN and LDT provide to different thalamic nuclei, and most notably those located in the midline, are in agreement with the notion that PPN and LDT maintain a clear functional topographical organization across many other targets (Mena-Segovia and Bolam, 2017). Our findings thus support the functional specialization of the PPN and LDT and open new directions to investigate their influence on thalamic circuits and their impact on adaptive behavior.

## Synthesis

Computational Neuroscience Model Code Accessibility Comments for Author (Required):

N/A

Significance Statement Comments for Author (Required):

N/A

Comments on the Visual Abstract for Author (Required):

N/A

## Author Response

The findings are of importance for efforts to understand the mechanisms of temporal integration and persistent firing. The manuscript presents an advancement in the current understanding of the oculomotor integrator circuit. In it, the authors detail differences in the recurrent excitation mechanism between the horizontal and vertical integrators. The results and the methods are well presented and thorough. The discussion, however, is very misleading and argues for differences in the putative mechanism that were not presented in the results. The idea of hub neurons made in the discussion can be supported by reconstruction the morphology of the neurons that were recorded. The authors claim that the time constants are short due to the lack of inputs from the contralateral side (because of the slice prep.) and cite key paper to support this claim. However, they omit citing another paper (Debowy et. al, 2011) that shows that midline lesions do not affect the time constants.

Major comments:

The paper by Saito and Sugimura presents evidence through slice physiology in the rodent model that a contributing synaptic mechanism for temporal integration at the Interstitial Nucleus of Cajal (INC), a key part of the neural integrator for vertical eye movements, is feedback through NMDA receptors. They also confirm their earlier findings that NMDA mediated inputs do not contribute significantly in the Nucleus Prepositus Hypoglossi (NPH), a key part of the neural integrator for horizontal eye movements, where instead Calcium-Activated Nonselective (CAN) cation currents play a more meaningful role. The experiments are well done and the work is presented in a straightforward manner. The findings are of importance for efforts to understand the mechanisms of temporal integration and persistent firing. I suggest a more careful revision of the discussion that makes claims that are supported by the evidence presented here.

Reply:

Thanks to the reviewer's suggestion, we have noticed that the statements in the previous version may provide the impression that the existence of hub neurons has already been clarified in the integrator networks. As the reviewer suggested, evidence of morphological and physiological connections to surrounding neurons is needed to claim the existence of hub neurons, and this evidence has not been obtained in the PHN or INC. Our previous study on the PHN (Saito and Yanagawa 2010) showed that the excitatory networks responsible for the sustained EPSC responses included neurons that did not express CP-AMPA receptors as well as neurons that expressed the receptors. This finding raises a possibility that some PHN neurons that express CP-AMPA receptors and some INC neurons that express NMDA receptors may play a role of hub neurons. We have modified the statements regarding hub neurons in order to not mislead readers (Page 22, line 1 -10).

A study using monkeys demonstrated that lesions of the commissural inhibitory networks of the neural integrators induced a dramatic reduction in the time constant of the integrators (Arnold and Robinson 1997). This finding suggests that positive feedback excitation through a mutual inhibition between the bilateral integrator regions is one of the mechanisms that maintain the activity of integrator networks. However, as the reviewer pointed out, negative evidence on positive feedback excitation has been obtained in goldfish studies. We have added this finding to the Discussion (Page 22, the last two lines).

Minor comments:

Please describe the expected damage from slice preparation to the dendritic arbor of cells in the NPH and INC.

Reply:

In the previous version, we stated that NMDA-dependent dendritic plateau depolarization of cortical pyramidal neurons may occur in INC neurons. However, the reviewer has expressed a concern about damage to the dendrites in our slice preparations. We agree with the reviewer that some dendrites of INC neurons may have been damaged or truncated during slicing. Therefore, whether dendritic plateau depolarization mediated via NMDA receptors indeed occurs in INC neurons remains an open question. We have added this statement to the text (Page 21, line 1 - 4).

In Figure 1, please plot the event rate vs time as well as the frequency and duration values as in Figure 2.

Reply:

In accordance with the reviewer's suggestion, we have added histograms showing the relationship between the event rate and the time to Figure 1.

The cobalt staining results need to be quantified and properly normalized by the total neuron number in the visualized region. Total counts could be acquired through a nuclear stain.

Reply:

To confirm the existence of neurons that express CP-AMPA receptors by another analysis in addition to the electrophysiological RI analysis, we performed a Co2+- uptake analysis. Our finding confirmed the existence of the neurons in both the PHN and INC.

In accordance with the reviewer's suggestion, we estimated the percentage of neurons that expressed CP-AMPA receptors. Because nuclear staining without an immunohistochemical procedure (for example, using DAPI) labels not only nuclei of neurons but also nuclei of other cells including glia, we performed Nissl staining using NeuroTrace (Invitrogen) to stain neurons. However, the Nissl staining was incomplete in sections in which the Co2+- uptake procedure was performed. Therefore, we performed Nissl staining using a section not exposed to the Co2+- uptake procedure of approximately the same location as the section exposed to the procedure. The neuron counts were performed in Co2+- uptake sections and Nissl-stained sections separately. In the PHN, there were 100.3 ± 58.5 (n = 3 sections) and 220.0 ± 38.2 (n = 2 sections) Co2+-labeled neurons and Nissl-stained neurons, respectively. In the INC, there were 48.7 ± 35.8 (n = 3 sections) and 118.0 ± 14.1 (n = 2 sections) Co2+-labeled neurons and Nissl-stained neurons, respectively. From these data, the percentage of Co2+-labeled neurons in the PHN and the INC were 45.6% and 41.2%, respectively. However, these values were obtained by an extremely rough analysis; therefore, we have not added them to the text to prevent these values from being taken out of context. We would like to develop a new method to precisely estimate the percentage of Co2+-labeled neurons in the PHN and INC in the future.
